# 

**Published:** 1872-10

**Authors:** 


### OUR PREMIUMS.

It will be observed, by referring to our premium list, which we have displayed upon one of the advertising pages of this number, that we are offering increased inducements to those who will assist us in enlarging our list of subscribers. We pay very liberal cash premiums, so that several of our agents are making from six to ten dollars a day, in soliciting subscribers to the BISTOURY. The subscription price is so small, and the journal so valuable to every household, that scarcely any trouble is experienced in securing patrons for it. We call attention to several new premiums which we offer, and hope our friends will exert themselves to secure subscribers to the BISTOURY, and good pay for themselves.

### BOOK NOTICES.

PARASITES, a little hand book upon animal and vegetable parasites of the human skin and hair, by B. Joy Jeffries, A. M., M. D., published by Alex. Moore, Boston, is a beautifully illustrated and plainly written treatise upon this subject. Price, $1.00.

PLAIN TALK ABOUT INSANITY.—This is the title of a work by T. W. Fisher, M. D., and treats upon the causes, forms, symptoms and treatment of mental diseases, with remarks on hospitals and asylums, and the medico legal aspect of insanity. Published by Alex. Moore, Boston. Price, $1.50.

SMALL-POX is the title of a little paper pamphlet treating upon the predisposing conditions and their treatment, of small-pox, by Carl Both. Alex. Moore, publisher, Boston. Price, 25 cents.

LIGHTS AND SHADOWS OF NEW YORK LIFE; OR, THE SIGHTS AND SENSATIONS OF THE GREAT CITY. A work descriptive of New York City in all its various phases. Its splendors and wretchedness; its high and low life; its marble palaces and dark dens; its attractions and dangers; its rings and frauds; its leading men and politicians; its adventures; its mysteries and crimes. By James D. McCabe, Jr.

The National Publishing Co., of Philadelphia, has just issued one of the most remarkable and attractive books of the day, bearing the above title. It is comprised in one large octavo volume of 850 pages, and illustrated with nearly 200 fine engravings of noted places, life and scenes in New York.

To all who contemplate visiting the great Metropolis, we cordially recommend it, both for its information and its powerful warnings against the dangers of the city. Those who cannot see New York for themselves will be in a great measure repaid for that privation by reading this work. It is published in both English and German; sold by subscription only, and the publishers want agents in every county.

### EXCHANGES.

PHILADELPHIA MEDICAL TIMES.—This splendid medical journal is constantly improving. It has the best contributors in this country. Published semi-monthly, by J. B. Lippincott & Co., Philadelphia, at four dollars a year.

DETROIT REVIEW OF MEDICINE is the title of a very neat monthly magazine, edited by Drs. L. Connor and A. B. Lyons, at Detroit, Mich. Terms, two dollars a year.

THE NORTH-WESTERN MEDICAL AND SURGICAL JOURNAL, edited by H. C. Hand, M. D. and H. H. Kimball, M. D., is the name of a very good journal published at St. Paul, Minn. Price, three dollars per annum.

GOOD HEALTH, a magnificent monthly magazine upon health matters, published by Alex. Moore, Boston, at two dollars a year.

Any of the above journals sent with the BISTOURY free, at publishers' price.

### SUBSCRIPTIONS RECEIVED.

In this column we will credit every subscription received during the last quarter. Subscribers will please notify us immediately if their payments are not duly credited. The State only is given—this, with the full name of the party subscribing, will be sufficient to indicate who is meant:

NEW YORK.—Thos White, E C Coryell, Capt E Wheeler, Dr G E Lawrence, Dr J H Gallinger, Rev R W Ripley, Dr H A Bolles, L L Barney, Rev Geo Brown, L P Barrows, Dr W H Cunningham, W F Ball, Dr Van Vechten, Dr W E Richards, W A Lawrence Henry Hobart, *Dr P Kent, Mrs Atherton, Dr S R Snook, Dr J P Cummings, Dr O V Northrup, Mrs L A Corrigan, Mrs O Kingsbury, Dr Mumford, Rev P L Kinney, Dr R Knight, Drs Mussey & Mills, O C Goodrich, R Chase, M Gregg, B Dusenberry, Dr Oustrander, Dr L M Bigalow, Dr B Moss, Mrs A Chamberlain, L S Bacon, Chas Morgan, T P Bradley, Dr A Dickinson.

PENNSYLVANIA.—Harrison Ross, Robert Bishop, E P Payne, Mrs L A Mackey, Mrs E C MClure, Dr Fishburn, Mrs Jas Pollock, Dr H C Lichtenthaler, Dr J Davidson, Dr J H McCarthy, Wm Stuart, †J Lowry Johnston, Mrs D M Johnston, Dr B Snevelley, Dr U V Long, Dr Simon Egglesby, Mrs S R Mayhew, B J Donaldson, M K Wright, Jno Fairman.

CONNECTICUT.—Dr Garry H Miner.

FLORIDA.—Mrs B Zuingin.

ILLINOIS.—Jno R Miller.

IOWA.—Dr F K Reinhold, Dr L O Dodge.

KENTUCKY.—Dr T L Newberry, Mrs C M Frost, L Kemberly, J R. Nichols, Dr J Bragg.

MISSOURI.—Dr J P Woods, Dr L M Howard, Mrs A Clemmens, W Williams, J S Salmon, Dr W Oglesby.

TENNESSEE.—Mrs J R Smith, Dr O P Kendrick.

NO PLACE.—Jas B Clark, Mrs Emma Mead.

NO NAME.—One dollar, post-marked *somewhere* in Pennsylvania.

* Club of twenty—one address.

† Paid up to April, 1873.

The above are subscriptions received up to Sept. 18th, the time of going to press.

THE BISTOURY,

ELMIRA, N. Y.

### A USEFUL PREMIUM.

For one hundred and ten subscribers to THE BISTOURY, at *Fifty Cents each*, amounting to $55,00, we will give a

### Grover & Baker Sewing Machine,

***Costing $55,00!***

Everybody knows that a GROVER & BAKER Machine is, so that it requires no puffing. We will guarantee them to be new, and in perfect and complete order.

For twenty subscribers, at fifty cents each, we will send to the getter up of the club, one of

### McAllister's New Microscopes,

The price of which is Five Dollars.

For seventy subscribers we will give as a premium a good, silver, hunting case, AMERICAN WATCH, worth $35,00.

Those who prefer to work for money, can do so according to our

### CASH LIST.

Examine it and learn *How to make from ten to twenty dollars a day!*

| | | | | | |
|---|---|---|---|---|---|
| For 100 | Subscribers and | $50,00 | you may | retain | $25,00 |
| " 50 | " | 25,00 | " | " | 10,00 |
| " 25 | " | 12,50 | " | " | 5,00 |
| " 10 | " | 5,00 | " | " | 2,00 |
| " 5 | " | 2,50 | " | " | 1,00 |

So that you get your premiums in SOLID CASH, instead of cheap books, worthless pictures, or "pinched beck" jewelry.

**THE WAY TO REMIT TO US.**

Collect $50 from 100 Subscribers. Then put $25 of it in your own pocket for your work and buy a Post Office order or draft with the other $25, and remit it to us. Also, send the names and address, (Post Office, County and State,) of each subscriber, PLAINLY WRITTEN, when they will receive THE BISTOURY by return mail, which will be an evidence to them that your contract has been faithfully performed. For fifty subscribers you will send us $15, you retaining $10, and so on, down through the list.

Women, who are worn out with sewing, throw down your needles, take a copy of THE BISTOURY and solicit subscriptions from your neighbors. You will find it a healthier and more profitable employment.

Post Masters can, without trouble, secure large lists at their offices, and make it pay better than their salaries.

Young men, out of employment, can earn a suit of clothes in one day, and make their canvassing pay them ever afterwards.

Boys and girls can canvass for THE BISTOURY,

**AND MAKE MORE MONEY**

than they ever dreamed of possessing before.

Try it, everybody. There is money in it, and

**You have your Pay in your own hands.**

Send all sums over $5 by Post Office Order or Draft, made payable to THAD S. UP DE GRAFF, M. D. Smaller sums may be sent in currency.

☞ Those who desire to get up Clubs, will have sample copies sent them FREE, by writing for them. ADDRESS,

**THE BISTOURY.**
**ELMIRA, N. Y.**

### AN ELEGANT PREMIUM!

The Young Folks' Rural, Two Chromos & The Bistoury,

ALL FOR

**ONE DOLLAR AND FIFTY CENTS.**

THE YOUNG FOLKS' RURAL is a rural and literary monthly journal, for young people of country and city. It is a beautifully printed, and elegantly illustrated sixteen page paper, brim full of entertaining stories, sketches, puzzles, plays, enigmas, charades, riddles, etc., etc., of surpassing interest to young people. We regard it as the very best youth's journal published in this country. It is published in Chicago, by H. N. F. Lewis, at $1.50 a year. Two elegant Chromos given to every subscriber.

We offer it together with Chromos and Bistoury, one year, for $1,50.

Address, **THE BISTOURY,**
ELMIRA, N. Y.

### THE BISTOURY GIVEN AWAY.

OUR TERMS FOR CLUBBING WITH OTHER JOURNALS.

We will cause any of the publications below to be sent together with THE BISTOURY, for one year, on the following terms:—

| | PUB. PRICE. | WITH BISTOURY. |
|---|---|---|
| American Agriculturalist | $1 50 | $1 50 |
| Scribner's Monthly | 4 00 | 4 00 |
| Peterson's Ladies' Magazine | 2 00 | 2 00 |
| iHome and Health | 1 50 | 1 50 |
| Little Corporal | 1 50 | 1 50 |
| Good Health | 2 00 | 2 00 |
| Atlantic Monthly | 4 00 | 4 00 |
| Western Rural | 2 00 | 2 00 |
| Young Folks' Rural | 1 00 | 1 00 |
| Peter's Musical Monthly | 3 00 | 3 00 |
| Eclectic Magazine | 5 00 | 5 00 |
| The Galaxy | 4 00 | 4 00 |
| Hearth and Home | 4 00 | 4 00 |
| Our Own Friends | 1 50 | 1 50 |
| Godey's Lady's Book | 3 00 | 3 00 |
| Wood's Household Magazine | 1 00 | 1 00 |
| The Children's Hour | 1 25 | 1 25 |
| Saturday Evening Post | 2 50 | 2 50 |
| Phrenological Journal | 3 00 | 3 00 |
| Medical Times, Philadelphia | 4 00 | 4 00 |
| The Aldine | 5 00 | 5 00 |
| Merry's Musem | 1 50 | 1 50 |
| Ballou's Monthly | 1 50 | 1 50 |
| Arthur's Home Magazine | 2 00 | 2 00 |
| Monthly Novelet | 2 00 | 2 00 |
| Our Young Folks | 2 00 | 2 00 |
| Ohio Farmer | 2 00 | 2 00 |
| Cultivator and Country Gentleman | 2 50 | 2 50 |
| New York Independent | 2 50 | 2 50 |
| American Union | 2 50 | 2 50 |
| Rural New Yorker | 3 00 | 3 00 |
| Saturday Night | 3 00 | 3 00 |
| Sadies' Repository | 3 50 | 3 50 |
| Harper's Magazine | 4 00 | 4 00 |
| " Bazar | 4 00 | 4 00 |
| " Weekly | 4 00 | 4 00 |
| Every Saturday | 5 00 | 5 00 |
| North American Review | 6 00 | 6 00 |
| Lippincott's Magazine | 4 00 | 4 00 |
| American Farmer's Advocate | 1 00 | 1 00 |

Forward the price of any of the above publications to our address, and you will receive both journals regularly, for one year. Address

**The Bistoury,** ELMIRA, N. Y.

